# Supplementary material for: Fusarium oxysporum effector clustering version 2: An updated pipeline to infer host range
Source: Front Plant Sci. 2022 Oct 19;13:1012688. doi: 10.3389/fpls.2022.1012688 (PMC9627151; doi:10.3389/fpls.2022.1012688)
Supplement: Supplementary file 2 [file DataSheet_1.pdf]

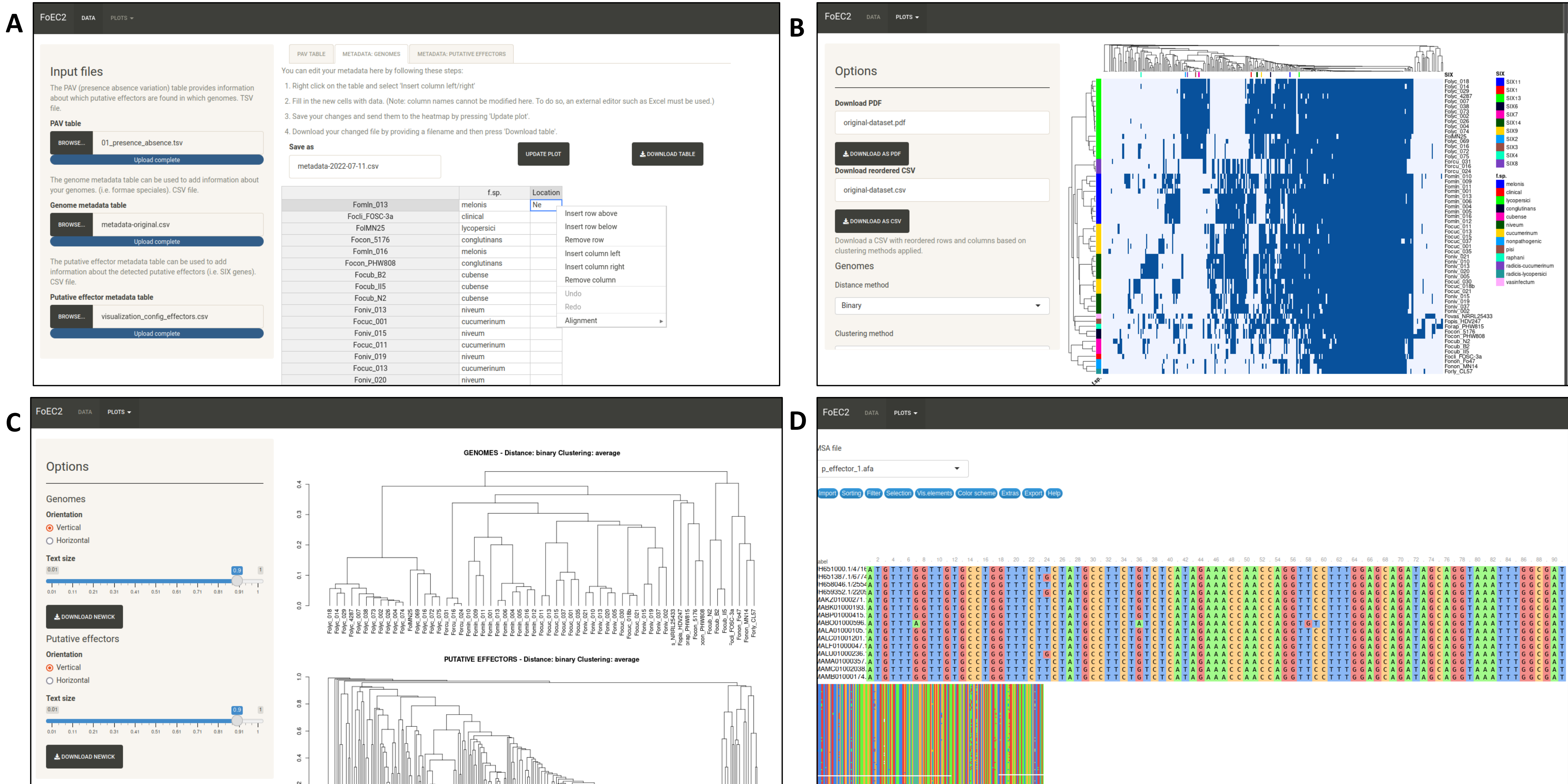

A
FoEC2
DATA
PLOTS
Input files
The PAV (presence absence variation) table provides information about which putative effectors are found in which genomes. TSV file.
PAV table
BROWSE...
01_presence_absence.tsv
Upload complete
The genome metadata table can be used to add information about your genomes. (i.e. formae speciales). CSV file.
Genome metadata table
metadata-original.csv
The putative effector metadata table can be used to add information about the detected putative effectors (i.e. SIX genes). CSV file.
Putative effector metadata table
visualization_config_effectors.csv
PAV TABLE
METADATA: GENOMES
METADATA: PUTATIVE EFFECTORS
You can edit your metadata here by following these steps:
1. Right click on the table and select 'Insert column left/right'
2. Fill in the new cells with data. (Note: column names cannot be modified here. To do so, an external editor such as Excel must be used.)
3. Save your changes and send them to the heatmap by pressing 'Update plot'.
4. Download your changed file by providing a filename and then press 'Download table'.
Save as
metadata-2022-07-11.csv
UPDATE PLOT
DOWNLOAD TABLE
f.sp.
Location
Insert row above
Insert row below
Remove row
Insert column left
Insert column right
Remove column
Undo
Redo
Alignment
B
Options
Download PDF
original-dataset.pdf
DOWNLOAD AS PDF
Download reordered CSV
original-dataset.csv
DOWNLOAD AS CSV
Download a CSV with reordered rows and columns based on clustering methods applied.
Genomes
Distance method
Binary
Clustering method
SIX
f.sp.
C
Options
Genomes
Orientation
Vertical
Horizontal
Text size
DOWNLOAD NEWICK
Putative effectors
GENOMES - Distance: binary Clustering: average
PUTATIVE EFFECTORS - Distance: binary Clustering: average
D
MSA file
p_effector_1.afa
Import
Sorting
Filter
Selection
Vis.elements
Color scheme
Extras
Export
Help
