# Supplementary figures and images for: Fusarium oxysporum effector clustering version 2: An updated pipeline to infer host range
Source: Front Plant Sci. 2022 Oct 19;13:1012688. doi: 10.3389/fpls.2022.1012688 (PMC9627151; doi:10.3389/fpls.2022.1012688)

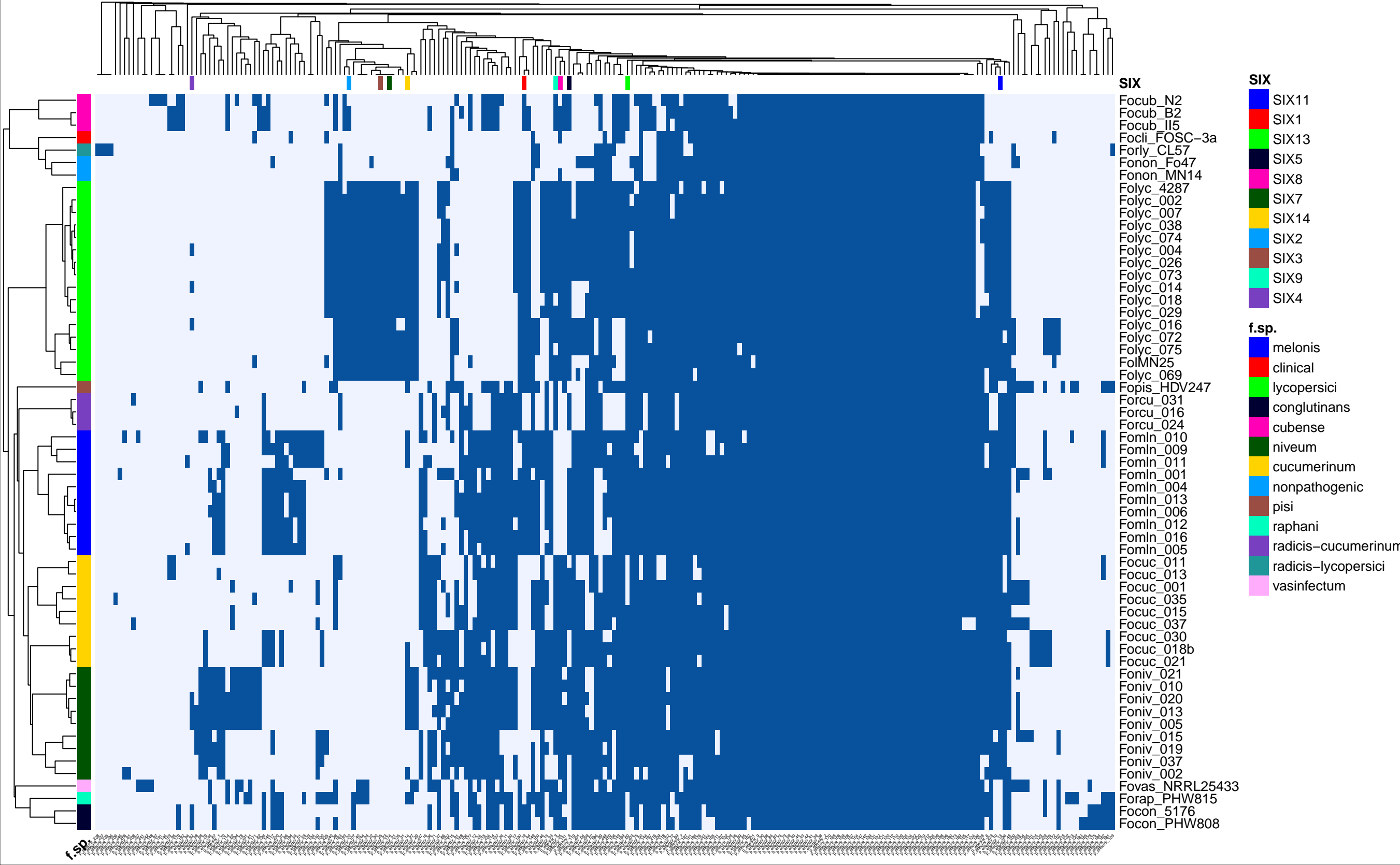

Supplement: Supplementary file 3 [file DataSheet_2.pdf]

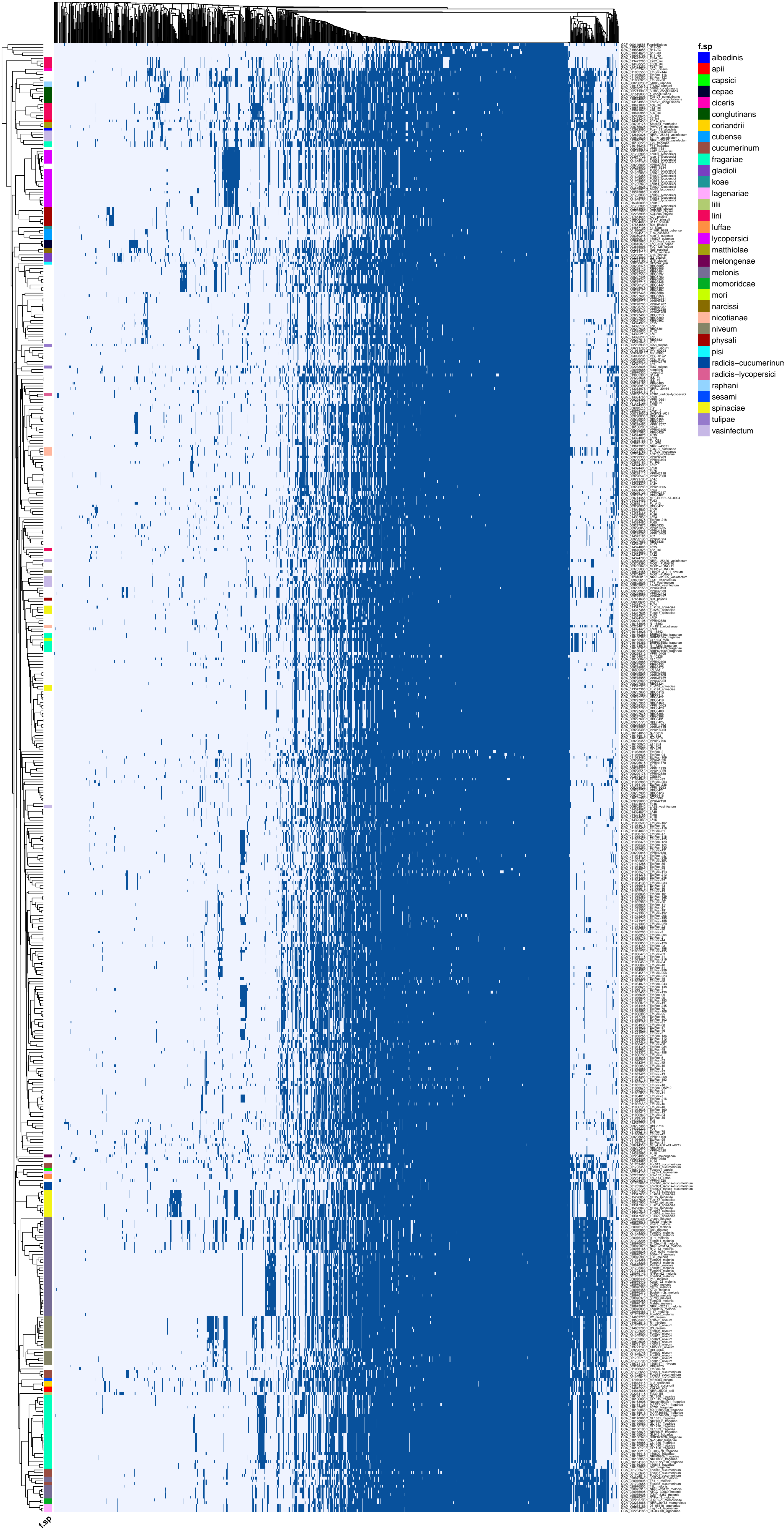

Supplement: Supplementary file 4 [file DataSheet_3.pdf]

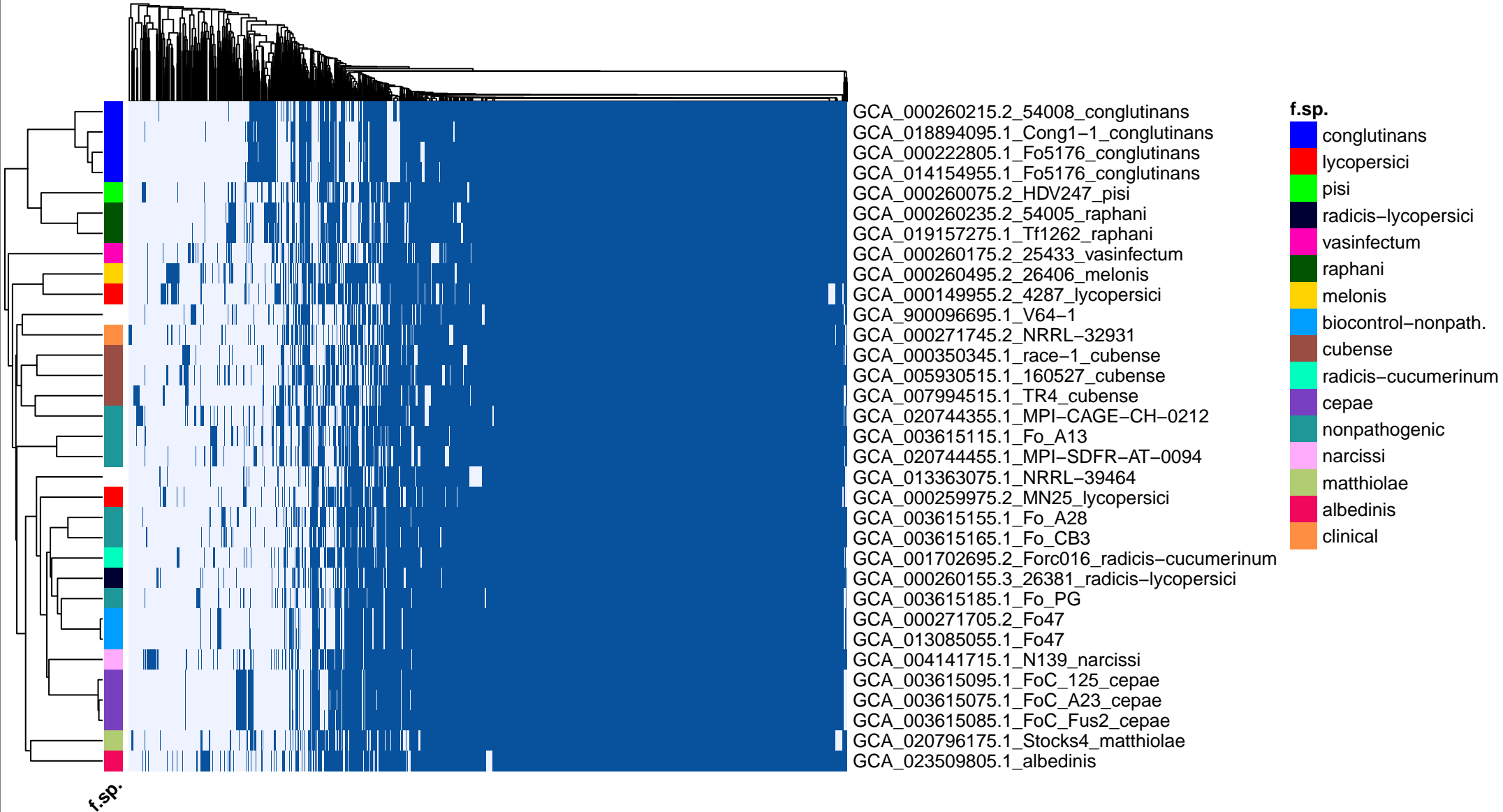

Supplement: Supplementary file 6 [file DataSheet_5.pdf]

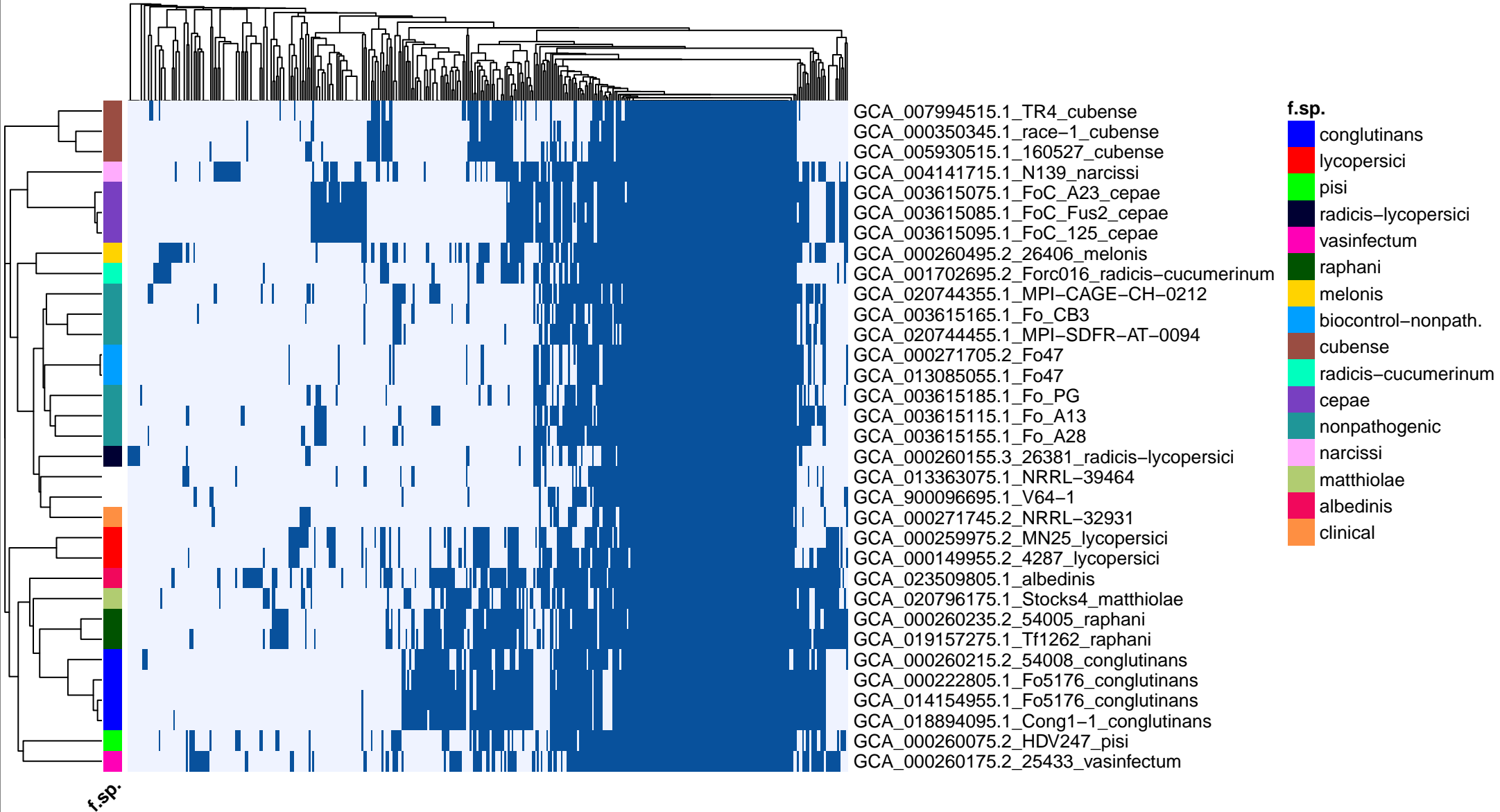

Supplement: Supplementary file 7 [file DataSheet_6.pdf]
