# Supplementary material for: Fusarium oxysporum effector clustering version 2: An updated pipeline to infer host range
Source: Front Plant Sci. 2022 Oct 19;13:1012688. doi: 10.3389/fpls.2022.1012688 (PMC9627151; doi:10.3389/fpls.2022.1012688)
Supplement: Supplementary file 5 [file DataSheet_4.pdf]

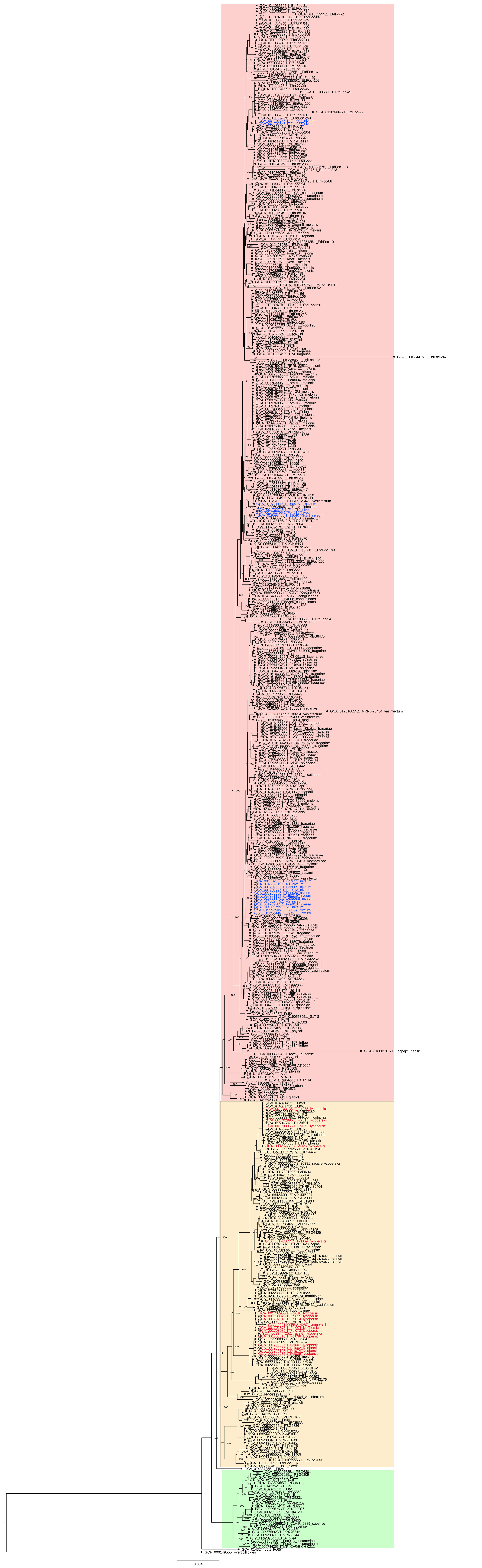
GCA_011034415.1_EtdFoc-247
GCA_012610825.1_NRRL-25434_vasinfectum
GCA_016801315.1_Focpep1_capsici
GCF_000149555_Fverticillioides
0.004
